# Introgression between *Anopheles gambiae* and *Anopheles coluzzii* in Burkina Faso and its associations with *kdr* resistance and *Plasmodium* infection

**DOI:** 10.1186/s12936-019-2759-1

**Published:** 2019-04-11

**Authors:** Mark J. Hanemaaijer, Hannah Higgins, Ipek Eralp, Youki Yamasaki, Norbert Becker, Oscar D. Kirstein, Gregory C. Lanzaro, Yoosook Lee

**Affiliations:** 1 Vector Genetics Laboratory, Department of Pathology, Microbiology and Immunology, School of Veterinary Medicine, UC Davis, Davis, CA 95616 USA; 2 German Mosquito Control Association (KABS), Speyer, Germany; 3 Centre for Organismal Studies, University of Heidelberg, Heidelberg, Germany

**Keywords:** *Anopheles*, Insecticide resistance, Gene flow, *Plasmodium*, Malaria vector, Burkina Faso

## Abstract

**Background:**

Insecticide resistance in *Anopheles coluzzii* mosquitoes has become widespread throughout West Africa including in Burkina Faso. The insecticide resistance allele (*kdr* or L1014F) is a prime indicator that is highly correlated with phenotypic resistance in West Africa. Studies from Benin, Ghana and Mali have suggested that the source of the L1014F is introgression of the 2L divergence island via interspecific hybridization with *Anopheles gambiae.* The goal of this study was to characterize local mosquito populations in the Nouna Department, Burkina Faso with respect to: (i) the extent of introgression between *An. coluzzii* and *An. gambiae*, (ii) the frequency of the L1014F mutation and (iii) *Plasmodium* infection rates.

**Methods:**

A total of 95 mosquitoes were collected from ten sites surrounding Nouna town in Kossi Province, Burkina Faso in 2012. The species composition, the extent of introgression in *An. coluzzii* mosquitoes and their *Plasmodium* infection rates were identified with a modified version of the “Divergence Island SNP” (DIS) genotyping assay.

**Results:**

The mosquito collection contained 70.5% *An. coluzzii*, 89.3% of which carried a 3 Mb genomic region on the 2L chromosome with L1014F insecticide resistance mutation that was introgressed from *An. gambiae*. In addition, 22.4% in the introgressed *An. coluzzii* specimens were infected with *Plasmodium falciparum*, whereas none of the non-introgressed (“pure”) *An. coluzzii* were infected.

**Conclusion:**

This paper is the first report providing divergence island SNP genotypes for natural population of Burkina Faso and corresponding *Plasmodium* infection rates. These observations warrant further study and could have a major impact on future malaria control strategies in Burkina Faso.

**Electronic supplementary material:**

The online version of this article (10.1186/s12936-019-2759-1) contains supplementary material, which is available to authorized users.

## Background

The malaria parasite, *Plasmodium falciparum*, continues to be a significant cause of illness and death in Burkina Faso (BF) [1]. In 2016, it was estimated that there were 7,890,000 malaria cases due to *P. falciparum* in Burkina Faso, 21,300 of which resulted in death [1]. Two of the main vector species implicated in the spread of malaria across BF are *Anopheles gambiae* and *Anopheles coluzzii* [2]. One of the malaria intervention strategies in the country has been the distribution of insecticide-treated bed nets (ITNs): ITN coverage increased from less than 40% in 2010 to over 60% in 2014 [1]. However, malaria cases in BF increased sharply in the period from 2010 to 2016 [1]. This apparent paradox may be explained by the high frequency of 2L chromosome introgression, which includes the knockdown resistance mutation variants (*kdr*), mutations (designated L1014F and L1014S) which have been associated with increased susceptibility to *Plasmodium* infection in individuals carrying this mutation [3].

Samples collected from the Nouna Department in 2010 showed that 64% and 50% of *An. gambiae* and *An. coluzzii*, respectively, possessed the L1014F insecticide resistance mutation [2]. Furthermore, the levels of L1014F mutation have remained relatively stable and high in the *An. gambiae* complex across BF, whereas the distribution and frequency of the L1014S mutation has significantly increased from < 10 to ~ 40% between 2008 and 2014 [4]. Previous studies show that *An. coluzzii* collected in Benin [5], Mali [6], and Ghana [7] acquired the L1014F mutation by introgression of a divergence island on the 2L chromosome. Nouna Department in BF is geographically located between Benin, Ghana, and Mali; therefore, similar patterns of introgression on the 2L chromosome in *An. coluzzii* are expected.

Previous studies determined introgression rates in BF based on 2 markers located close to the L1014F mutation [8]. However, whole genome sequencing of introgressed *An. coluzzii* reveal that the genomic island of divergence, a genomic region of 3 Mb near the centromere on the 2L chromosome, which is highly diverged between *An. coluzzii* and *An. gambiae*, was introgressed from *An. gambiae* to *An. coluzzii* [6, 7]. Recently, it has also been shown that introgressed *An. coluzzii* mosquitoes have higher *Plasmodium* infection rates [3, 9, 10]. Mitri and his co-workers suggested that this phenotype is not caused by the L1014S or L1014F mutation, but most likely by the serine protease *ClipC9* gene that is located on the 3 Mb genomic region that is introgressed [3]. The ‘Divergence Islands SNP’ assay [11] was improved to simultaneously identify species, introgression of the chromosome 2L 3 Mb genomic region, insecticide resistance, and *Plasmodium* infection. The goal of this study is to determine the introgression status and corresponding *Plasmodium* infection rates in the Nouna Department, BF.

## Methods

### Sample collection and DNA extraction

*Anopheles gambiae* and *An. coluzzii* were collected from ten villages within a 30 km radius in the Nouna Department, BF in 2012 (Table 1). DNA was extracted from head/thorax tissue using the QIAGEN Biosprint 96 system with QIAGEN blood tissue reagents following established protocols [12, 13]. A modified version of the “Divergence Island SNP” (DIS) assay [11, 14] was used to distinguish *An. gambiae*, *An. coluzzii* and introgressed *An. coluzzii*. *Anopheles gambiae* and *An. coluzzii* are differentiated in the DIS assay using SNP markers on the X, and 2L and 3L chromosomes [11]. All SNPs are located within three unlinked pericentromeric genome regions known as the islands of divergence [15, 16]. *Anopheles coluzzii* samples were considered “pure” when the majority (> 15/18) of DIS markers had *An. coluzzii* specific genotypes. An *An. coluzzii* sample with more than two gambiae-specific markers is considered “introgressed”. This is consistent with previous studies [11, 14]. The L1014F mutation was not considered a species-specific marker. *Plasmodium* infection rates were assessed by including mitochondrial DNA makers of *Plasmodium* species that can distinguish *Plasmodium ovale*, *Plasmodium malariae*, *Plasmodium vivax*, and *P. falciparum* (see Additional file 1). Some of these markers are from the *Anopheles* multi-detection assay [17]. To accommodate all these in a single multiplex assay, some of previous DIS markers (1039-358, 0407-337 SNPs) were replace with 00819-1180 SNP. The final set of markers used for the new DIS assay and the related metadata are provided (see Additional file 1).

**Table 1:** Sampled villages around Nouna and the corresponding species distribution

Collection sites	Latitude	Longitude	Collection year	*An. arabiensis*	“Pure” *An. coluzzii*	Introgressed *An. Coluzzii*	*An. gambiae*	N
Biron Badala	12.6072	− 3.5455	2012	1	0	9	0	10
Biron Marka	12.5739	− 3.6336	2012	1	1	8	0	10
Bourasso	12.6337	− 3.7126	2012	5	0	5	0	10
Cisse	12.8960	− 3.7355	2012	1	2	7	0	10
Goni	12.5792	− 3.9627	2012	0	0	10	0	10
Kamadena	12.3759	− 3.5297	2012	3	2	4	1	10
Kansara	12.6907	− 3.7842	2012	1	2	7	0	10
Kodougou	12.5183	− 3.6078	2012	3	0	7	0	10
Konkuini	12.6717	− 3.7854	2012	3	0	7	0	10
Tissi	12.8492	− 3.7323	2012	1	1	3	0	5
Total				19	8	67	1	95

## Results and discussion

### Insecticide resistant *Anopheles coluzzii* show patterns of introgression

**Fig. 1:**
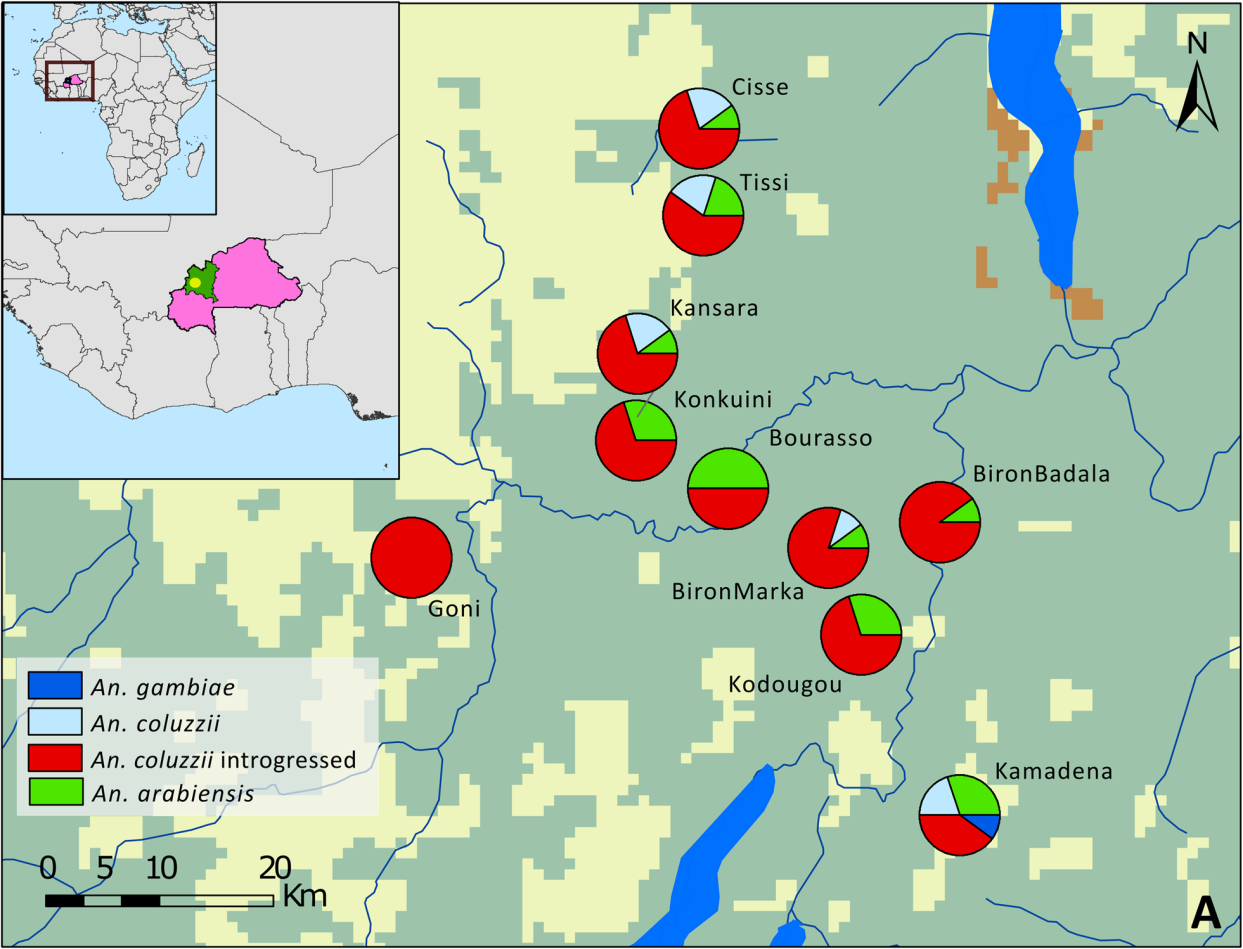
Species distribution around Nouna, BF. Map colours represent different ecotypes: yellow represents cultivated and managed areas. Green represents croplands, shrubs or herbaceous covers. Brown represents deciduous shrubs and blue represent inland fresh waters

A total of 95 *Anopheles* mosquitoes were collected from villages within 30 km radius in Nouna Department, BF. The species composition based on genotyping is listed in Table 1 and illustrated on the map in Fig. 1a. The raw genotyping results are listed (see Additional file 2). *Anopheles coluzzii* is the most abundant vector species in the majority of villages. In the village of Bourasso equal numbers of *An. arabiensis* and *An. coluzzii* were found. In Kamadena, the location furthest away from Nouna town, one *An. gambiae* sensu stricto (s.s.) specimen was found, together with *An. arabiensis* and *An. coluzzii*. The relative abundance of *An. arabiensis* (20%) observed near Nouna differs from previous reports that *An. arabiensis* was absent in that location [2]. This observation suggests that *An. arabiensis* can increase in abundance after successful introduction of insecticide-treated bed nets, as observed in many locations throughout sub-Saharan Africa [18–20], possibly because it is less affected by insecticide-treated bed nets compared with *An. gambiae* [20]. Furthermore, the relative abundance of *An. gambiae* observed (1.1%) is significantly lower than that reported by Namountougou and co-workers, who found *An. gambiae* at a frequency of 23.3% in their survey [2]. The overall pattern is consistent with a collapse of the local *An. gambiae* s.s. population, apparently being replaced by introgressed *An. coluzzii* carrying the L1014F (*kdr*) mutation, as observed in Mali [21].

The majority of specimens we collected were *An. coluzzii* (75 out of 95). Of those, 89.3% were individuals that showed introgression of the chromosome 2L genomic island of divergence [15]. The 2L introgressed *An. coluzzii* includes the L1014F mutation originating from *An. gambiae* (Table 2). One heterozygous L1014F mutation in a “pure” *An. coluzzii* individual was observed, which based on the genotyping assay, showed no further history of introgression with *An. gambiae* (all *An. coluzzii* specific SNPs present on the 2L chromosome). Similar patterns of introgression of the 2L chromosome were observed in Mali [6, 21], Ghana [7] and Guinea Bissau [11]. In addition, 3 out of 19 *An. arabiensis* mosquitoes (16.8%) were heterozygous for the L1014S mutation (*kdr*-e) (Table 2). This variant, originally found in East Africa, is now increasingly found in West Africa [22, 23].

**Table 2:** L1014 genotype distribution in *Anopheles* populations in villages around Nouna, BF

Collection sites	*An. arabiensis*			“Pure” *An. coluzzii*			Introgressed *An. coluzzii*			*An. gambiae*		
	+/+	+/E	E/E	+/+	W/+	W/W	+/+	W/+	W/W	+/+	W/+	W/W
Biron Badala	1	0	0	0	0	0	0	2	7	0	0	0
Biron Marka	1	0	0	1	0	0	0	1	7	0	0	0
Bourasso	4	1	0	0	0	0	0	2	3	0	0	0
Cisse	1	0	0	2	0	0	0	5	1	0	0	0
Goni	0	0	0	0	0	0	0	4	6	0	0	0
Kamadena	3	0	0	2	0	0	0	1	3	0	0	1
Kansara	1	0	0	1	1	0	0	2	5	0	0	0
Kodougou	3	0	0	0	0	0	0	3	4	0	0	0
Konkuini	1	2	0	0	0	0	0	3	4	0	0	0
Tissi	1	0	0	1	0	0	0	1	2	0	0	0
Total	16	3	0	7	1	0	0	24	42	0	0	1

+/+ represents the homozygous susceptible genotype, W/+ represents the L1014F heterozygous genotype and W/W represents the homozygous L1014. E allele represent L1014S mutation

### Introgressed *Anopheles coluzzii* are more likely to be infected with *Plasmodium falciparum*

From 95 *Anopheles* specimens sampled, 18 were positive for *P. falciparum* (19%, Table 3). This infection rate is comparable with those observed in Cameroon [24], Kenya [25] and the Democratic Republic of Congo [26]. Infections by *P. ovale*, *P. vivax*, or *P. malariae* were not observed among the samples.

**Table 3:** *Plasmodium falciparum* infection counts in villages around Nouna, BF

Collection sites	*An. arabiensis*		“Pure” *An. coluzzii*		Introgressed *An. coluzzii*		*An. gambiae*		Total infected
	Uninfected	Infected	Uninfected	Infected	Uninfected	Infected	Uninfected	Infected	
Biron Badala	1	0	0	0	9	0	0	0	0
Biron Marka	1	0	1	0	5	3	0	0	3
Bourasso	3	2	0	0	4	1	0	0	3
Cisse	1	0	2	0	7	0	0	0	0
Goni	0	0	0	0	6	4	0	0	4
Kamadena	3	0	2	0	3	1	1	0	1
Kansara	1	0	2	0	6	1	0	0	1
Kodougou	2	1	0	0	5	2	0	0	3
Konkuini	3	0	0	0	4	3	0	0	3
Tissi	1	0	1	0	3	0	0	0	0
Total	16	3	8	0	52	15	1	0	

The infection rate in introgressed *An. coluzzii* (N = 67) was 22.4%, whereas none of the non-introgressed, “pure” *An. coluzzii* (N = 8) were infected with *Plasmodium* (Table 3). The SNP genotyping assay can detect *P. falciparum*, *P. vivax*, *P. malariae* and *P. ovale*, but all infections detected were with *P. falciparum*. The difference in *Plasmodium* infection rates between “pure” and introgressed *An. coluzzii* were not statistically significance using Fisher’s Exact Test (p = 0.345) due to the low number of pure *An. coluzzii* mosquitoes. Power calculations based on this data indicate that further studies with increased sample size (N > 190) would likely be required to provide sufficient statistical rigor.

Although differences in the *Plasmodium* infection rate between introgressed and non-introgressed, “pure” *An. coluzzii* is not statistically significant, there is evidence in the literature that introgressed *An. coluzzii* may be more susceptible to *Plasmodium* infection [3]. Silencing the serine protease ClipC9 gene, which is located within the 2L introgressed genomic region, does significantly increase parasite numbers in *An. coluzzii* laboratory colony mosquitoes [3]. One may also suspect that increased longevity due to increased insecticide tolerance could affect the rate of *Plasmodium* infection in introgressed *An. coluzzii.* Moreover, whether this pattern would hold over multiple collections across different seasons remain to be determined. Further research is required to understand the true impact of this gene in natural *An. coluzzii* populations.

## Conclusion

The results indicate that introgressed *An. coluzzii* is the primary malaria vector in the Nouna Department of BF. These populations carry the L1014F and L1014S insecticide resistance mutations and also have higher *Plasmodium* infection rates compared to the other *Anopheles* populations observed in the region. These observations warrant further study and could have a major impact on future malaria control strategies in BF.

## Additional files

**Additional file 1.** SNP information on the DIS assay.

**Additional file 2.** Raw genotyping data of the collected mosquitoes around Nouna, BF.
